# Neural Correlates of the Perception for Novel Objects

**DOI:** 10.1371/journal.pone.0062979

**Published:** 2013-04-30

**Authors:** Hao Zhang, Jia Liu, Qinglin Zhang

**Affiliations:** 1 Key Laboratory of Cognition and Personality, Ministry of Education, School of Psychology, Southwest University, Chongqing, China; 2 State Key Laboratory of Cognitive Neuroscience and Learning, Imaging Center for Brain Research, Beijing Normal University, Beijing, China; University of Minnesota, United States of America

## Abstract

Perception of novel objects is of enormous importance in our lives. People have to perceive or understand novel objects when seeing an original painting, admiring an unconventional construction, and using an inventive device. However, very little is known about neural mechanisms underlying the perception for novel objects. Perception of novel objects relies on the integration of unusual features of novel objects in order to identify what such objects are. In the present study, functional Magnetic Resonance Imaging (MRI) was employed to investigate neural correlates of perception of novel objects. The neuroimaging data on participants engaged in novel object viewing versus ordinary object viewing revealed that perception of novel objects involves significant activation in the left precuneus (Brodmann area 7) and the right visual cortex. The results suggest that the left precuneus is associated with the integration of unusual features of novel objects, while the right visual cortex is sensitive to the detection of such features. Our findings highlight the left precuneus as a crucial component of the neural circuitry underlying perception of novel objects.

## Introduction

There are numerous novel objects around human beings. The perception for novel objects is a more important type of perception whereby people have an exceptional visual experience (e.g., seeing an original painting, admiring an unconventional construction, and using an inventive device). Previous studies have found that novelty processing affects subsequent cognitive or affective processes including memory, thinking, emotion and decision-making [1], [2], [3], [4], [5]. Some research has suggested that novelty processing involves neural networks in the prefrontal and posterior association cortices, medial temporal lobe, hippocampus, and substantia nigra [6], [7], [8], [9], [10], [11], [12], [13]. However, novel stimulus materials in these studies were pictures of real objects that were not familiar or showed to participants before the experiment.

Distinct from perception of ordinary objects, perception of novel objects involves the integration of unusual features of a novel object to identify what the object is. Such novel objects include characteristics of originality and appropriateness [14], [15], [16]. The originality of the objects refers to original qualities based on unusual features and their association within novel objects. The appropriateness is about useful or adaptive quality of novel objects in relation to the situation or the constraint [17], [18], [19], [20], [21], [22]. Therefore, perception of such novel objects incorporating both originality and appropriateness is different from the previous experiments [2], [5], [12], [13], [23], [24]. Recently, Stoppel et al. [23] employed Mandelbrot-fractals as novel stimuli to explore the influence of spatial attention on neural activity of novel responses. In this case, novel fractal pictures were novel for the participants, but lacked semantic content so that no appropriate quality was available. These stimuli were not suitable for investigating perception of novel objects. Therefore, prior studies of novelty processing without considering appropriate quality or controlling original quality well have not directly targeted neural substrates of perception of novel objects.

**Figure 1:**
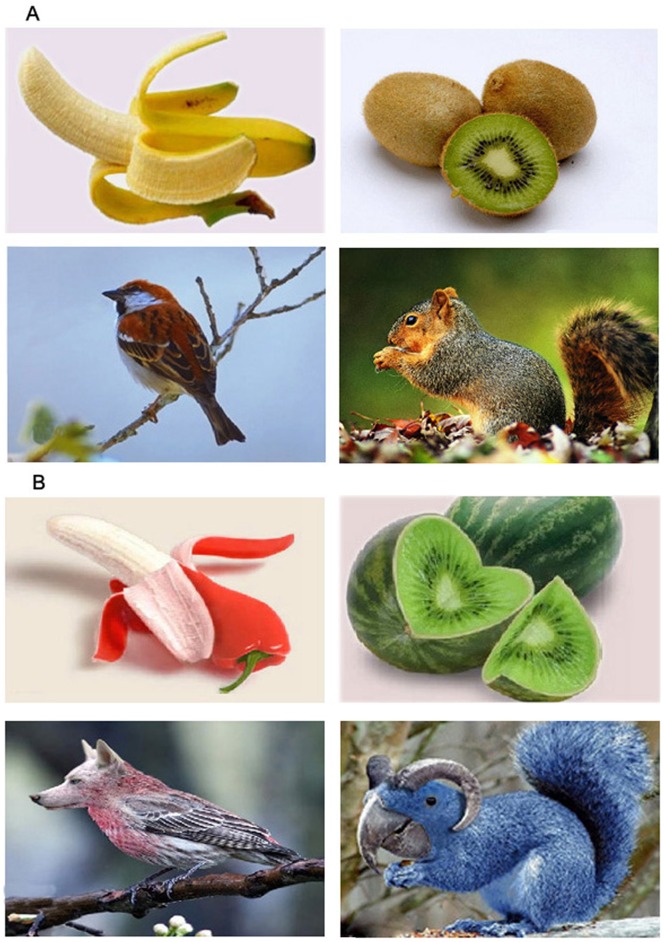
Examples of experimental materials. (A) Stimuli were used in ordinary object viewing tasks. (B) Stimuli were used in novel object viewing tasks.

The purpose of the present study was to identify brain regions associated with perception of novel objects, and differences in neural activity patterns between perception of novel objects and perception of ordinary objects. To reveal neural bases of perception of novel objects, we employed three visual perception tasks – an ordinary object viewing task, a novel object viewing task, and a baseline task – during scanning to map neural activity patterns underlying perception of ordinary objects, novel objects, and geometric patterns. The ordinary objects consisted of known animal or plant stimuli. The novel objects consisted of animal or plant stimuli, these objects are not existent in the world but these hybrid images were based on adaptive functional features of new species of biological beings recently discovered by biologists. These hybrid images are the biological bodies formed by the combination of distinct features of some animals or plants. The formed biological bodies are not existent in the world, so this ensures the original quality of these stimuli. Meanwhile, the combination of diverse functional features from different animals or plants makes a combined biological body possess more functional features than one of these animals or plants. The functional diversity in the combined body optimizes its appropriate quality. For example, at the left top in Fig. 1B there was a species: the internal soft part of the species was a banana, and the skin of the species was a red pepper. It is obvious that this is a new species relative to a banana or red pepper. Moreover, in the same species the skin is useful for a vegetable and internal part is useful for a fruit. As a consequence, these biological beings changed by functional features ensure both original quality and appropriate quality in relation to the situation or the constraint. Therefore, such stimuli met the criteria for the characteristics of novel objects. The baseline consisted of simple squares. Each of these stimuli was presented in visual perception tasks as a picture in a two-dimensional space.

Early visual processing occurs in the occipital cortex. Separate routes go to the temporal lobes and the parietal lobes. The ventral pathway is involved in objects identification and semantics [25]; the dorsal pathway, among other functions, is involved in spatial organization of the stimuli [26], [27]. Due to specific task requirements in the present study, neural mechanisms of perception should vary as a function of novel versus ordinary objects identification. Are there common components in neural pathways shared for perception of novel objects and perception of ordinary objects? What are differences between the two types of perception in the ventral and dorsal pathways? Based on a key constituent of perception of novel objects regarding the integration of unusual features of novel objects, and prior neuroimaging findings indicating the precuneus has a central role in highly integrated tasks [28], we hypothesized that after early visual processing in the occipital cortex, the precuneus in the dorsal pathway might be crucial to neural networks underlying the integration of unusual features of novel objects.

## Materials and Methods

### Participants

Eighteen college students (eight males, ten females, mean age 20.3 years, range 17–23 years) participated in the experiment as paid subjects. All were right-handed and none had a history of neurological or psychiatric mental problems. The study was approved by the Institutional Human Participants Review Board of the University Imaging Center for Brain Research, and written consent was obtained from all participants.

### Design and Materials

Three different conditions were used to investigate the neural correlates for perception of novel objects: an ordinary object viewing task, a novel object viewing task, and a baseline task. The ordinary object viewing task featured 40 pictures of regular animals and plants, while the novel object viewing task included 40 pictures of animals and plants that have never existed in the world. These original and appropriate biological bodies were formed by the combination of distinct features of different animals or plants (Fig. 1). The 80 pictures with moderate difficulty of viewing in two types of tasks were chosen from 160 pictures after the assessment by ten students who did not participate in the experiment. The rating for the difficulty of ordinary object identification was averaged 4.2 and the rating for the difficulty of novel object identification was averaged 4.7 in terms of a 7-point rating scale ranging from 1 (very easy) to 7 (very difficult). The baseline task consisted of six squares. 20 pictures of the baseline were dispersed among stimulus trials of novel objects and ordinary objects. The presentation of experimental stimulus trials was conducted in an event-related design.

In the scanner, stimulus trials of these tasks were presented in random order, and each stimulus trial lasted six seconds. Within experimental conditions, participants were required to view the displayed objects and patterns as soon as possible, and indicated whether they were identified successfully or not by pressing one of two buttons on a keypad, or press any button for baseline trials. To control for neural activation of the brain associated with motor action, nine participants responded with the index finger of their right hand and others responded with the index finger of their left hand. Immediately after scanning, participants were asked to complete a self-report questionnaire. The “items” in the questionnaire were the same stimuli as those displayed during scanning. The participants were required to recall and write down what these stimulus objects were during the scanning process.

### MRI Acquisition

MRI data were collected by a 3 Tesla Siemens MAGNETOM Trio. Participants laid supinely with their heads comfortably fixed by belt and foam pads to reduce head movement. Earplugs were used to dampen scanner noise. Visual stimuli were presented through a projector onto a screen in the bore of the scanner. Participants viewed the stimuli through a mirror mounted to the head coil. Behavioral responses were recorded by pressing buttons. High-resolution T1-weighted images were acquired for each participant to provide anatomical reference (1×1×1 mm^3^). Functional MRI data were collected using a T2* weighted gradient-echo echo planar imaging (EPI) sequence. In each volume, thirty-two slices (4-mm-thick) were acquired axially, interleaved slice mode to cover the whole brain. Data were recorded in a single session, and a total of 300 volumes were acquired with a repetition time (TR) of 2000 ms, an echo time (TE) of 30 ms, a flip angle of 90 degrees, field of view (FOV) of 200×200 mm, acquisition matrix of 64×64, and spatial resolution of 3×3×4 mm^3^.

### Data Analyses

Statistical Parametric Mapping (SPM2) [29] was employed for preprocessing and statistical analyses of imaging data with Matlab 6.5 (Mathworks). Slice timing correction was done before spatial processing due to acquisition of imaging data with interleaved slice mode. The functional image volumes were spatially realigned to reference volume, and then normalized to the standard brain template from the Montreal Neurological Institute [30] using nonlinear basis functions [31]. The images were spatially smoothed by an 8-mm full-width half-maximum (FWHM) isotropic Gaussian kernel [32]. Low-frequency drift in the BOLD signal was removed by a high-pass filter set at 128 s of cosine functions.

Based on the preprocessing of imaging data, individual analysis of imaging data was performed using the general linear model. The BOLD signal was modeled as a canonical hemodynamic response function. The contrasts of interest were examined after estimation of condition effects at each voxel. Our comparisons included perception of ordinary objects compared with geometric perception, perception of novel objects compared with geometric perception, and perception of novel objects compared with perception of ordinary objects. Each contrast produced a statistical parametric map of the *t* statistic (finally converted into *Z* values). The resulting activations were computed by a voxel-wise intensity threshold of *P*<0.05 using a correction of multiple comparisons via the family-wise error (FWE) [33], and a cluster size of a minimum of twenty contiguous voxels. Brain regions were estimated from Talairach and Tournoux [34] following adjustments for differences between MNI and Talairach coordinates.

## Results

### Behavioral Data

Reaction time and completion rate in the stimulus object viewing tasks were recorded during scanning. There were significant differences in reaction times for viewing novel objects [M = 3041 ms (SEM = 157)], ordinary objects [M = 1763 ms (SEM = 124)], and baselines [M = 954 ms (SEM = 83)]. *F* (2, 17) = 181, *P*<0.001. Post-hoc tests showed that differences were significant between novel and ordinary objects, and between novel objects and baselines. In addition, a significant difference in completion rate emerged between novel objects [87% (SEM = 4.5)], ordinary objects [99% (SEM = 0.4)], and baselines [98% (SEM = 0.8)]. *F* (2, 17) = 4, *P*<0.05, with post-hoc tests revealing significant differences between novel objects and ordinary objects, and between novel objects and baselines. This pattern suggested that perception of novel objects involves the integration of unusual features of novel objects, rather than familiar features of ordinary objects or simple features of geometric patterns. That is, such integration of unusual features in perception of novel objects takes longer time than that in perception of ordinary objects.

To examine what participants identified in viewing objects during scanning, we used a questionnaire with pictures of all stimulus objects, requesting participants write down by recalling them immediately after scanning. In the completed questionnaires, the participants wrote relevant names of these stimulus objects. For example, the participants wrote Chinese characters “辣蕉” (a peppery banana) for the novel object at the left top in Fig. 1B. It was interpreted as an original and useful species which was both vegetable red pepper and fruit banana. About the novel object at the left bottom in Fig. 1B, the participants wrote Chinese characters “狼鸟” (a wolf bird), and they interpreted this was an original and adaptive species which could fly and eat meat. It is obvious that these names were invented by the participants. Theses names never existed or said before in the real world, and were suitable for the novel objects. Such names reflected that participants detected and integrated unusual features of novel objects during identifying what novel objects were. In contrast, names of ordinary objects existed or said before in everyday life, hence implicating perception of ordinary objects based on the retrieval of features and their relations within regular objects from long-term memory. The questionnaire data suggested that participants experience different perceptual processes during distinctive object viewing tasks.

### Neuroimaging Data

**Figure 2:**
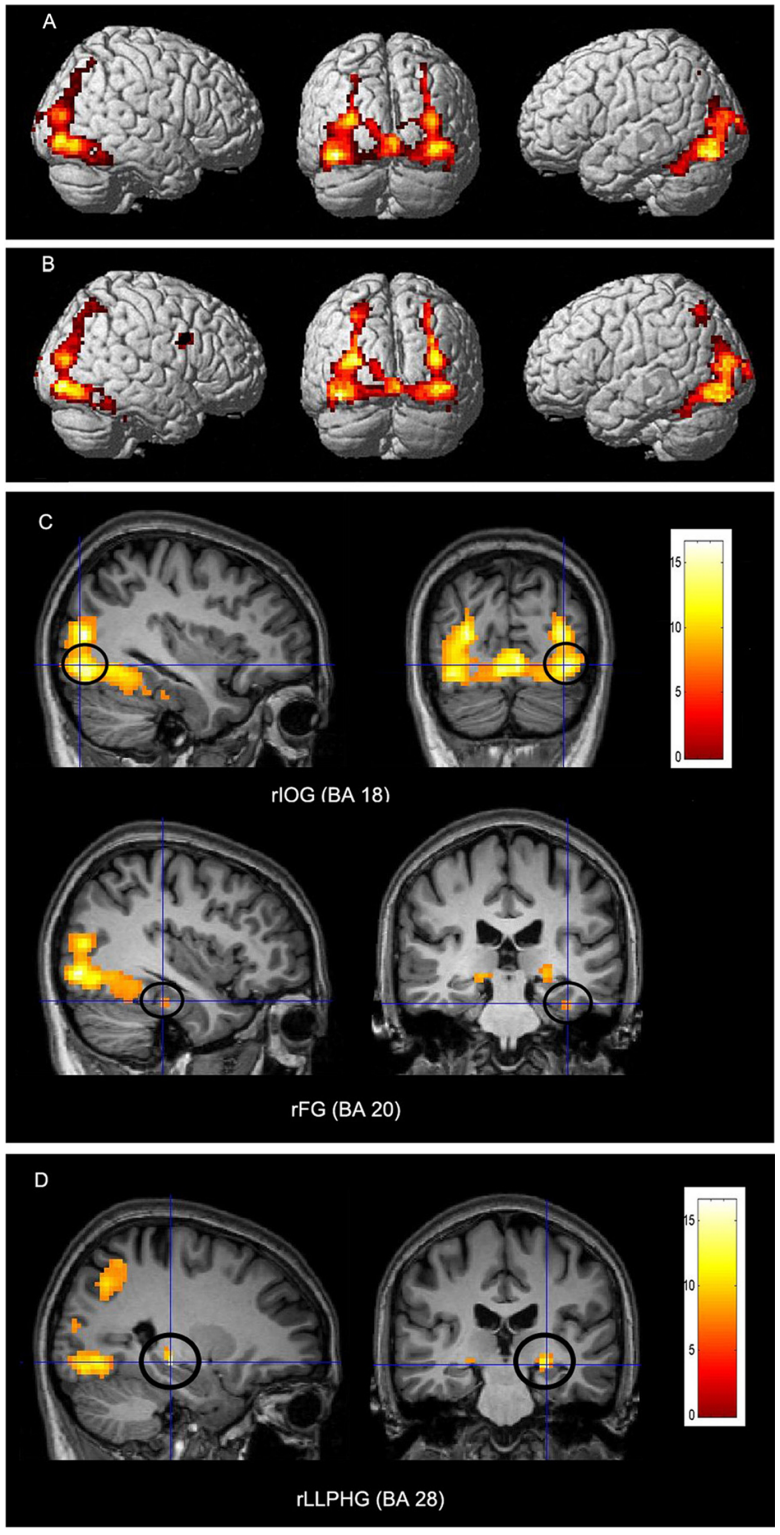
Cortical activity during perception of ordinary objects and novel objects. (A) and (C) Cortical activations associated with perception of ordinary objects compared to baseline. Two regions of activation in the ventral pathway were the right inferior occipital gyrus (rIOG) and the right fusiform gyrus (rFG). (B) and (D) Cortical activations associated with perception of novel objects compared to baseline. The activation region in the ventral pathway was the right limbic lobe parahippocmpal gyrus (rLLPHG). A and B are lateral and back view. C and D are sagittal and coronal sections. The significance thresholds are *P*<0.05 FWE-corrected (the family-wise error) with an extent threshold of 20 contiguous voxels. Functional maps shown at sagittal and coronal sections are overlaid on the T1-weighted images.

A series of comparisons of experimental conditions was conducted to examine the neural network underlying perception of novel objects. The first comparison of ordinary object viewing versus the baseline condition revealed that the cognitive operation of perception of ordinary objects involved significant activations in the right inferior occipital gyrus (BA 18: *x* = 35, *y* = −81, *z* = 0), left inferior occipital gyrus (BA 19: *x* = −38, *y* = −70, *z* = −4), right middle occipital gyurs (BA 19: *x* = 35, *y* = −77, *z* = 17), right fusiform gyrus(BA 20: *x* = 38, *y* = −30, *z* = −18), and right sub-lobar lateral geniculum (*x* = 26, *y* = −26, *z* = −3) (Fig. 2 A and C).

The second comparison of novel object viewing versus the baseline condition revealed the cognitive operation of perception of novel objects to involve significant activations in the right inferior frontal gyrus (BA 9: *x* = 44, *y* = 6, *z* = 21), left superior parietal lobule (BA 7: *x* = −26, *y* = −58, *z* = 44), right middle occipital gyrus (BA 19: *x* = 35, *y* = −77, *z* = 17), left middle occipital gyrus (BA 19: *x* = −29, *y* = −80, *z* = 17), right limbic lobe parahippocampal gyrus (BA 28: *x* = 26, *y* = −26, *z* = −6) (Fig. 2 B and D).

**Figure 3:**
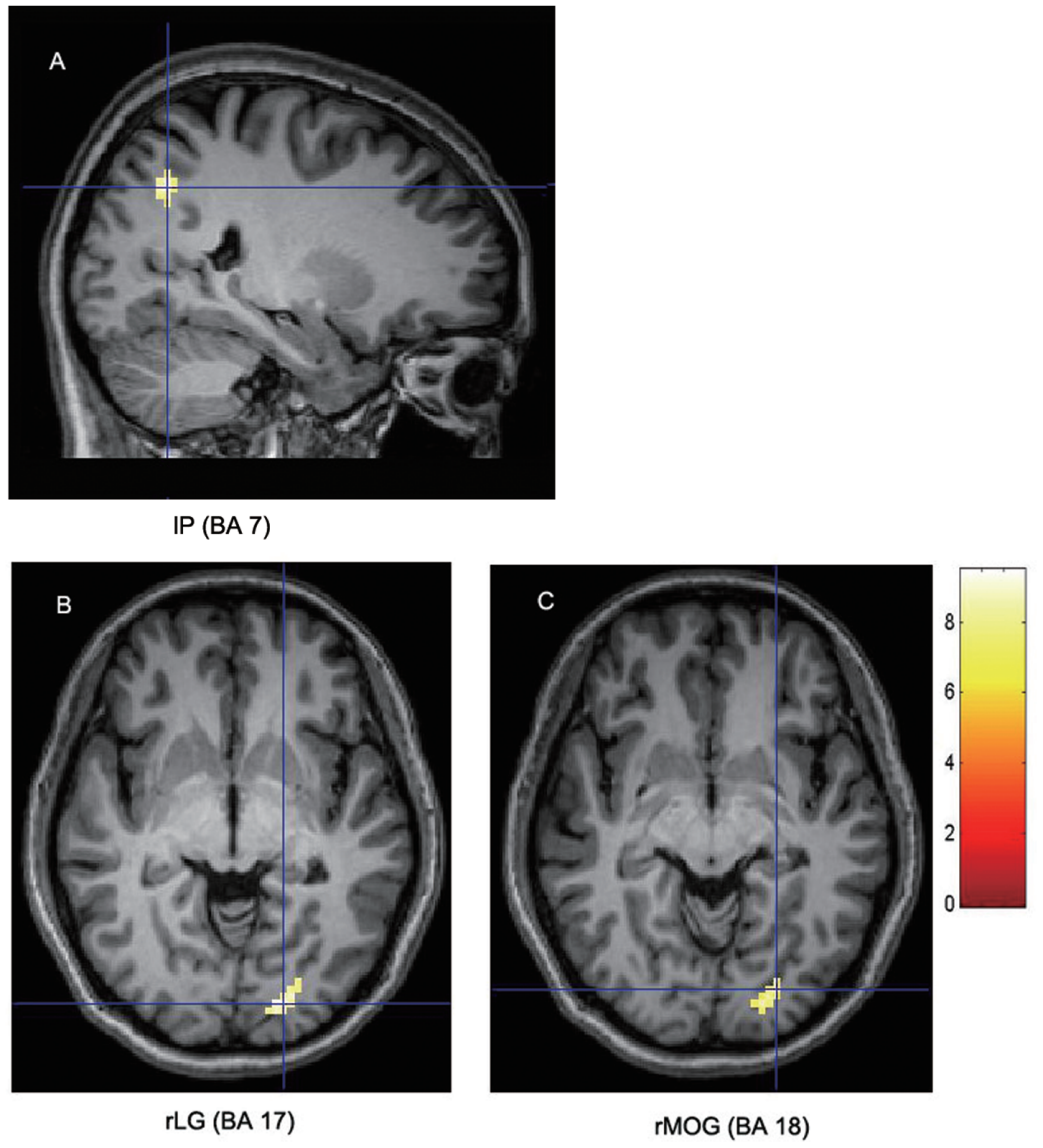
Significant activations elicited by perception of novel objects. Regions showing significant activations were associated with perception of novel objects compared with ordinary objects (masked inclusively with both novel and ordinary conditions versus geometric condition). (A) Activation at BA 7 in the left precuneus (lP). (B) Activation at BA 17 in the right lingual gyrus (rLG). (C) Activation at BA 18 in the right middle occipital gyrus (rMOG). The significance thresholds are *P*<0.05 FWE-corrected with an extent threshold of 20 contiguous voxels. Functional maps shown at sagittal and axial sections are overlaid on the T1-weighted images.

As the cognitive subtraction principle indicated, neural activity patterns of direct comparison between the two viewing tasks might reflect crucial neural components of perception of novel objects. Therefore, the third comparison of novel object viewing versus ordinary object viewing (masked inclusively with novel and ordinary object tasks minus geometric pattern tasks) was undertaken and revealed the cognitive operation of perception of novel objects to be associated with significant activations in the left precuneus (BA 7: *x* = −23, *y* = −62, *z* = 39), right lingual gyrus (BA 17: *x* = 20, *y* = −87, *z* = 0), and right middle occipital gyrus (BA 18: *x* = 26, *y* = −81, *z* = −3) (Fig. 3 A, B, and C). The reverse comparison of ordinary object viewing versus novel object viewing (masked inclusively with novel and ordinary object tasks minus geometric pattern tasks) revealed significant activations in the right cuneus (BA 23: *x* = 8, *y* = −75, *z* = 9), left lingual gyrus (BA 18: *x* = −5, *y* = −72, *z* = 6), and left cuneus (BA 17: *x* = 0, *y* = −81, *z* = 9) (Table 1).

**Table 1:** Coordinates of activation peaks.

Regions activated	BA	*x*	*y*	*z*	*Z*-score
**Ordinary-baseline**					
Occipital lobe					
Right inferior occipital gyrus	18	35	−81	0	6.87
Left inferior occipital gyrus	19	−38	−70	−4	6.86
Right middle occipital gyrus	19	35	−77	17	6.86
Temporal lobe					
Right fusiform gyrus	20	38	−30	−18	5.09
Subcortical regions					
Right lateral geniculum		26	−26	−3	5.85
**Novel-baseline**					
Frontal lobe					
Right inferior frontal gyrus	9	44	6	21	5.50
Parietal lobe					
Left superior parietal lobule	7	−26	−58	44	6.89
Occipital lobe					
Right middle occipital gyrus	19	35	−77	17	6.82
Left middle occipital gyrus	19	−29	−80	17	6.60
Subcortical regions					
Right parahippocampal gyrus	28	26	−26	−6	6.45
**Ordinary-novel**					
Occipital lobe					
Right cuneus	23	8	−75	9	5.81
Left lingual gyrus	18	−5	−72	6	5.29
Left cuneus	17	0	−81	9	5.69
**Novel-ordinary**					
Parietal lobe					
Left precuneus	7	−23	−62	39	5.47
Occipital lobe					
Right lingual gyrus	17	20	−87	0	5.52
Right middle occipital gyrus	18	26	−81	−3	5.18

BA indicates Brodmann area. *x*, *y*, and *z* represent position in Talairach coordinate space.

## Discussion

The present study employed the stimulus object viewing task and functional MRI to identify neural bases of perception of novel objects. Functional MRI data revealed that perception of novel objects involves significant activation in the left precuneus (BA 7) and the right visual cortex (BAs 17 and 18), when participants were engaged in novel object viewing compared with ordinary object viewing. These results suggest that the left precuneus (BA 7) is associated with the integration of unusual features of novel objects and the right visual cortex is sensitive to the detection of unusual features of novel objects.

Perception is to explain how we attach meaning to sensory information we receive. Perception of novel objects involves the integration of unusual features, in addition to sensory information input and retrieval of prior information in perception of ordinary objects. This is a reason why perception of novel objects takes longer time than perception of ordinary objects. Imaging data analyses for perception of novel objects compared with the baseline condition showed activation of widely distributed areas of the brain (Table 1). This supported contentions that multiple regions of the brain are recruited for perception of novel objects. Moreover, the analysis indicated that neural activity related to perception of novel objects is different from that of perception of ordinary objects in the ventral pathway (Fig. 2 A, B, C, and D), albeit there were a few common areas of activation. Meanwhile, neural activity related to perception of ordinary objects in the ventral pathway (Fig. 2 C) replicated prior studies of regular object perception [35], [36]. These distinct patterns in the ventral pathway reflected essential differences between perception of novel objects and perception of ordinary objects. Perception of ordinary objects demands participants to recognize familiar features of ordinary objects and retrieve prior existing associations regarding these features from long-term memory. Different from perception of ordinary objects, perception of novel objects requires participants to detect and integrate unusual features of novel objects, in addition to recognition of familiar features of the objects and retrieval of prior existing associations among the features. Furthermore, a direct comparison of novel versus ordinary object viewing allows us to identify key neural components of perception of novel objects [37]. This revealed significant activation in the left precuneus (BA 7) of the dorsal pathway and the right visual cortex (BAs 17 and 18) of the ventral pathway.

The precuneus or the medial extent of BA 7 corresponds to the medial aspect of the posterior parietal lobe, bounded anteriorly by the marginal part of the cingulate sulcus, posteriorly by the parietooccipital sulcus, and inferiorly by the subparietal sulcus [38], [39]. The functions of this long-neglected region have generated increasing interest. A recent resting-state fMRI study [40] across humans and monkeys found that the precuneus plays important functional roles: the anterior sector has a sensorimotor role, the posterior sector supports connections with the visual cortex, the central precuneus has cognitive associative functions. Studies of white matter track have revealed that the precuneus has abundant connections with other brain areas including the frontal, temporal, occipital, and other parietal cortices [41], [42], [43], [44]. In addition, there are subcortical connections to the thalamus, striatum, claustrum and brainstem [45], [46], [47]. It is clear that the precuneus is the hub of reciprocal cortical and subcortical connections and plays a key role in a wide variety of integrated tasks [28].

Earlier studies of the precuneus were from patients with optic ataxia. The patients are impaired in reaching and grasping under visual guidance. Impairment reflects poor hand-eye movement coordination, rather than motor, somatosensory, or visual field disorders [48]. Researchers assume such lesions are found at the superior parietal lobe or intraparietal sulcus [49]. Recent research on stroke and tumor patients with optic ataxia has shown that the medial parietal cortex controls visually guided hand movements [50]. One functional MRI study investigated bimanual and unimanual movements along different directions, and trajectories of movement of the left and right hand were spatially incompatible [51]. Bimanual movements require additional coordination effort to break away from intrinsically favored mirror movements, and integrate movements of both hands into a new spatial pattern under visual supervision. Their results showed that the execution of spatially complex bimanual movements compared with unimanual movements involved the anterior cingulate cortex and dorsoanterior precuneus. The researchers claim the anterior cingulate suppresses intrinsically-favored tendencies, and the precuneus contributes to control the additional coordination or integration of eye and bimanual movement. Based on this evidence, the precuenus (BA7) plays a crucial role in integration of eye and hand movement.

Beyond processing of visual and hand movements, recent studies suggest the precuneus has a role in integrating the implementation of high-order cognitive functions. In a functional MRI study [52], reasoning tasks related to visual, spatial, and other relations were presented acoustically via headphones to exclude the effect of visual verbal stimuli. The premise of each reasoning problem was about a relation such as “cleaner/dirtier” or “smarter/dumber” between the dog, the cat, and the ape. Conclusions were drawn by connecting various propositions or mental images in the premises. In the process, logic rules were applied to mental representations of the inferential process [53], [54], [55]. Furthermore, the integration of propositions or mental images with logic rules becomes a crucial component of deductive reasoning. The researchers found that all types of reasoning problems evoked activity in the bilateral precuneus and other regions compared to the rest interval. The results suggested that activation of the precuneus is dedicated to the integration of propositions or mental images in deductive reasoning. Consistent evidence from brain lesions and functional MRI has implicated the precuneus (BA 7) in various types of highly integrative tasks. Findings from the present study add new knowledge to the precuneus functions associated with the integration of unusual features of novel objects in perception of novel objects.

Besides that, we notice that novel object stimuli also possibly induced more attention and eye movements in visual processing, although we did as possible as match the shape, size, and structure between novel object stimuli and ordinary object ones in selecting experimental stimulus. As previous functional imaging and lesion case showed, shifting attention involves the superior parietal cortex [56], [57], [58]. In addition, eye position modulated neuronal activity in parietal cortex [59]. It is possible that shifting attention and eye movements additionally contributed to neural activity in the precuneus. Therefore, the neural activity in the precuneus in our study was evoked by the integration of unusual features of novel objects, and it was affected by shifting attention and eye movement.

Perception of novel objects in the present study also induced neural activity in the primary visual cortex (BA 17) and extrastriate visual cortex (BA 18). Neurons in these regions are involved with the detection of visual features for constructing complex visual representations [60], [61], [62]. Our experiment revealed activation of BAs 17 and 18 visual regions in perception of novel objects. This suggests that the visual cortices BA 17 in the lingual gyrus and BA 18 in the middle occipital gyrus are sensitive to detect unusual visual features of novel objects. This is consistent with neuroimaging studies related to visual perception [63], [64], [65], [66], [67]. For example, in Stoppel et al.’s [23] fMRI study, the lingual gyrus was found to process novel events when stimuli appear outside the focus of spatial attention. They suggested that the lingual gyrus is linked to the detection of perceptual novelty. Jung et al. [68] used structural MRI to examine cortical thickness of the brain related to the creative process in young participants. They found that BA 18 is correlated with creative tasks. Consonant with previous findings, our study further implicated the role of BAs 17 and 18 in the detection of unusual visual features of novel objects. Beyond this point, it is unlikely to happen that the two regions were recruited to attention and eye movement in our study. If cortical activation at BAs 17 and 18 was evoked by attention and eye movement when participants viewed the novel objects compared with the ordinary objects, there should be increasing activation at BAs 17 and 18 that was evoked by attention and eye movement when participants viewed the novel objects compared with baseline stimuli. However, in the results of the present study (Table 1), when participants viewed the novel objects compared with baseline stimuli, there was not activation at BAs 17 and 18 regions. Thus, it is not possible that the activation at BAs 17 and 18 regions was related to the potential difference in attention and eye movement between the two main tasks.

In summary, the present study investigated neural mechanisms underlying perception of novel objects. The key comparison of novel versus ordinary object viewing tasks revealed significant activation of the left precuneus (BA 7) in the dorsal pathway and the right visual cortex (BAs 17 and 18) in the ventral pathway. Our results suggested the left precuneus (BA 7) is associated with the integration of unusual features of novel objects, and the right visual cortex (BAs 17 and 18) in the ventral pathway is sensitive to the detection of unusual features of novel objects. Thus, the present study reveals that the left precuneus (BA 7) is a crucial component of the neural circuitry underlying integration processing in perception of novel objects. This finding sheds light on neural mechanisms of the perception for creative products.
